# Urban slum housing quality, and its public health implications in Nigeria: a case of urban slum residents in Enugu metropolis, South East, Nigeria

**DOI:** 10.1186/s12889-024-20764-7

**Published:** 2024-11-20

**Authors:** Salomey N. Ogbonna, Casmir N. Ochie, Elias C. Aniwada

**Affiliations:** 1 Department of Community Medicine, University of Nigeria College of Medicine, Ituku-Ozalla, Enugu, Nigeria; 2 Department of Community Medicine, University of Nigeria Teaching Hospital, Ituku-Ozalla, Enugu, Nigeria

**Keywords:** Housing and health, Public health, Implications, Urban slums, Enugu metropolis

## Abstract

**Introduction:**

Housing remains a strategic social determinant of health. In Sub-Saharan Africa, most urban dwellers live in slums with attendant health implications. This study assessed the housing conditions of the slums of Enugu metropolis and the public health implications.

**Methods:**

This is a community-based cross-sectional study of 459 slum residents of the Enugu metropolis. Ethical clearance was obtained from the UNTH, Health Research Ethics Committee (HREC). Data was collected using a pretested semi-structured interviewer-administered questionnaire and an observational checklist. Data was analysed using IBM SPSS version 23. Data were summarised using mean and standard deviation, frequency and proportion as appropriate and presented in tables and figures. The chi-square test was used for association at p-value ≤ 0.05 significance.

**Results:**

The mean age (SD) of the respondents was 32.93(12.34) years. A higher proportion (*N* = 180, 39.2%) were 18-27years, females (*N* = 282, 61.4%), married (*N* = 297, 64.7%), attained secondary education (*N* = 273, 59.5%), Igbo (*N* = 453, 98.7%), and self-employed (*N* = 327, 71.2%). They demonstrated good knowledge of standard housing specifications (*N* = 231, 50.3%) and the effects of housing conditions on health (*N* = 297, 64.7%). Also, most lived in a one-room apartment (*N* = 201, 43.8%) and cooked in a separate kitchen (*N* = 150, 32.7%) with a gas cooker as the major source of heat supply for cooking (*N* = 249, 54.2%). Sixty-three (13.7%) of the respondents didn’t have access to suitable toilet facilities. A higher proportion, (*N* = 171, 37.3%) used pipe-borne water, and electricity as the major source of light (*N* = 447, 97.4%). The most prevailing health condition was malaria/fever (*N* = 258, 97.4%). Despite having pests and rodents-infested dwellings, only (*N* = 156, 34.0%) had insecticides in the house. Equally, (*N* = 132, 28.8%) of them lived with broken floors.

**Conclusions:**

Despite good knowledge of the public health implications of poor housing, most dwellings remained substandard and unhealthful with associated prevalent health conditions.

**Recommendation:**

There is a need for a health campaign against the poor living conditions in the slums.

**Supplementary Information:**

The online version contains supplementary material available at 10.1186/s12889-024-20764-7.

## Introduction

Housing is the second most important essential need of man after food. People spend a reasonable amount of time in the house, the sick and the children even spend more. Housing in its entire ramification is more than a shelter and it embraces all social services and utilities that lead to worthy living [1, 2]. The importance of housing to health came into a discussion over a century ago and more so it has been noted that not just housing affects health but also the neighbourhood where the housing structure was situated [2]. Several attempts were made to define housing and differentiate it from healthful housing and home [3]. An age-long strong relationship has been identified to exist between housing and health [2, 4]. Housing is a strong social determinant of health and the quality of housing has major consequences for people’s health [4–6]. Qualities and features of housing may determine the physical, social and mental well-being of residents. With the mobility of labour, rural-urban migration, population growth and urbanization, challenges in housing become more difficult and it is progressively worsening, especially in urban areas. More so with the associated projected doubling of world urban population by 2050 it will worsen as this will create increased demand for housing globally [3].

The principal function of a house is to protect us from the elements and requires that a home provide not only shelter, but also privacy, safety, and reasonable protection of our physical and mental health. In contrast, a living facility that fails to offer these essentials through adequately designed and properly maintained interiors and exteriors cannot be termed “healthful housing” [2]. On the contrary, poor housing has been linked to a myriad of problems individually and collectively [4]. Aside from constituting a nuisance to the inhabitants and neighbourhood, disease outbreaks like all the water-related diseases, unsafe conditions which can lead to falls and mental ill health like anxiety were a few of the possible issues among others [1, 7]. The housing conditions are progressively getting worse, daily as a result of the rapidly increasing population of people living and working in the urban area leading to housing scarcity and unaffordability [3, 5].

The basic principles of healthful housing like guiding the fundamental needs cannot be compromised as a result. These fundamental needs include physiological and psychological needs, protection against disease, protection against injury, protection against fire and electrical shock, and protection against toxic and explosive gases [2, 8, 9]. Housing first and foremost functions as the physical protection it offers man and his domestic companion against cultural hazards in his physical environment [1, 2, 10]. The physiologic needs are protection from the elements, a thermal environment that will avoid undue heat loss, a thermal environment that will permit adequate heat loss from the body, an atmosphere of reasonable chemical purity, adequate daylight illumination and avoidance of undue daylight glare, direct sunlight, adequate artificial illumination and avoidance of glare, protection from excessive noise, and adequate space for exercise and for children to play. More so, the fundamental Psychological needs for healthy housing are adequate privacy for the individual, opportunities for normal family and community life, opportunities for normal community life, facilities that make possible the performance of household tasks without undue physical and mental fatigue, facilities for maintenance of cleanliness of the dwelling and of the person, possibilities for aesthetic satisfaction in the home and its surroundings, and concordance with prevailing social standards of the local community. In addition to the psychological value of privacy, repeated studies have shown that lack of space and quiet due to crowding can lead to poor school performance in children [2]. Crowding has also been identified as a significant risk associated with respiratory diseases, gastroenteritis and diarrhoeal diseases, poor mental health outcomes including stress, sleep disorders and poor educational attainment [11].

There is strong evidence between housing hazards and unintentional injuries, often affecting children [11]. Risks of injury inside the home include those relating to structural failure of the dwelling, fall and trip hazards, risks of electrocution and fire risks whereas indoor air quality is also affected by the types of finishing, furnishing, adhesives and coatings used in the building and this is further impacted by tobacco smoking by occupants or infiltrations of tobacco smoke coming from neighbouring units [11]. Dampness and mould are potential health risks and no level of mould growth is considered safe for health [11]. Inappropriate noise levels can lead to auditory and non-auditory effects on health [11]. This can result from the home or neighbourhoods. The following conditions were noted to significantly influence disease conditions reported and they include the indoor temperature controls/ventilation, the building condition, environmental quality and crowding. The disease conditions reported were asthma, pneumonia, bronchitis and cough were identified as the major determinants of cough [10, 12].

On the verge of reducing these untoward consequences of unhealthful housing, housing laws evolved. Housing laws are as old as humans and have evolved through several stages keeping pace with development [2]. The World Health Organisation (WHO) has also published, harmonized and integrated housing policies and regulations [11]. Health was then recognized as a core component of that policy.

Over a billion live in homes that are not only inadequate but are also harmful to health [5]. Excess winter deaths due to cold housing were estimated at 38 200 per year (12.8/100 000) in 11 selected European countries, and with improved housing standards, 47% of the occupants reported fewer sick building syndrome symptoms, fewer cases of mould, pests, inadequate ventilation and stuffiness in the green homes [11]. A high proportion of the global population, especially in low-income settings, continues to live in housing developed via informal mechanisms (including informal settlements (slums) and unauthorized developments [11]. There has been a 40% reduction in hospitalizations for diseases typically linked to poor housing following improvement in housing [11]. Each year, injuries occurring at home result in an estimated 4 million emergency department visits and 70,000 hospital admissions in the US and the contributing factors implicated included structural features in homes, including steep staircases and balconies, lack of safety devices such as window guards and smoke detectors, and substandard heating system [6]. In Sub-Saharan Africa, 71.8% of urban dwellers live in slums, the highest proportion in the world and the proportion of urban dwellers in Nigerians living in slums is about 75%^5^ Several authors reported that so many housing units in the rural areas were devoid of modern/adequate facilities, many of which were built with mud materials, poor housing and environmental condition, inefficiency of infrastructure and public services [3]. Multiple house problems have been linked to diseases and disability [4]. The quality of the environment in most urban centres in Nigeria is noted to be quite deplorable not talk of the slums [3].

The United Nations Human Settlements Programme (UNHS) defines a slum as a wide range of low-income settlements and/or poor human living conditions, which include the vast informal settlements that are quickly becoming the most visual expression of urban poverty [6]. A slum household is one lacking one or more of the following indicators access to improved water, access to improved sanitation facilities, insufficient living area, lack of structural quality and durability of dwellings and security of tenure [13]. Moreover, a slum area is largely inhabited, by people who have migrated from other areas [13]. One in eight people lives in slums globally and in total, around a billion people live in slum conditions today [14]. Nigeria has squatter settlements which are characterized by uncontrolled substandard temporary dwellings, poor sanitary conditions, dilapidated structures, and a high occupancy ratio [13]. Furthermore, some urban slums in Chile have been described as housing the poorest of the poor, the unemployed, the unskilled, and illiterate and often the alcoholics, the vagabonds and the delinquents [13]. In many poor countries they exhibit high rates of disease due to unsanitary conditions, malnutrition, and lack of basic health care [13].

Nigeria’s urban slum areas harbour over fifty per cent (53.3%) [15] of her population, yet the slum areas are the most neglected in terms of social welfare and health services provision relatively. This study explored the housing conditions and the gaps in healthful housing. Therefore, this study aims at the assessment of housing conditions and their health implications among the slum residents of Enugu metropolis, Enugu State, Nigeria.

## Methodology

This study was carried out in an urban slum of Enugu State metropolis. Enugu State is one of the 36 states of Nigeria and the metropolis is made up of three local government areas (LGAs). The state is located in the Southeastern geopolitical zone of Nigeria where it shares borders with Benue, Kogi, Anambra, Ebonyi, Imo and Abia states. Enugu lies between Latitudes 6.4413° North and Longitudes 7.4988° East [16] with tropical climates [17] and a land mass of 7,161 km^2^ with a population of 3,267,837 persons [18]. It is a densely populated area with a population density of 615.1 persons per square kilometre [19]. The current metro area population of Enugu in 2023 is 847,000, representing a 3.29% increase from 2022 [20].

The state operates a ward-based, 3-tier level of healthcare delivery (the primary, secondary and tertiary). Within this hierarchical arrangement, higher-order centres are expected to receive referrals from the lower-order centres.

The state has more than six urban slums found in the three LGAs of Enugu metropolis. In Enugu State, the recognized major slums consist of the central section of Ogui urban, Ogbete Colliery Camp, the Iva Valley coal Camps, Abakpa Nike, Ogui urban (Onu Asata), Iva Valley Quarters, Alfred Camp, China town, Udi siding, Uwani central, and Asata [21]. The slum lacks considerable infrastructural facilities like a good road network, and pipe-borne water, though there may be the presence of a primary health facility. They are densely populated and most of them are artisans, traders, daily-paid and migrant workers. The housing structures are not adequate and substandard.

The urban area is bequeathed with wonderful housing superstructures with most of the needed facilities for good physiological, physical and psychological needs unlike what is usually seen in the slum where the structures lack one or more of the attributes associated with healthful housing [22].

### Study Design and Population

this was a descriptive household cross-sectional survey using a questionnaire to assess housing conditions, public health regulations on housing and their health implications among residents in Enugu urban slum, Nigeria. The study was among adult household residents of all Enugu urban slums aged 18 years and above excluding residents who relocated to the urban slum but have not been residents for up to six months, visitors to the slums and those who were too ill to participate in the study.

### Sample size determination

Four hundred and fifty-nine residents partook in this study. The minimum sample size was determined using the sample size determination formulae for descriptive cross-sectional study [23].

$$n = \left( {{z^2}pq} \right)/{d^2}$$

where; **z** is the confidence level at 95% taken as 1.96, and **p** is the proportion of respondents (60%) of the respondents who knew that some conditions of their houses could affect their health in Benue, North Central Nigeria [5]. **q** is the complementary probability (1 – p) = 1-0.600 = 0.4 **d** is the desired precision of the study set at 0.05.

*N* = 1.962^**2(**^**(0.6) (0.4)/0.05**^**2**^**= 369.5 approx. 370;** 10% non-response rate = 37.

Then the minimum sample size is 370 + 37 = 407 respondents but was rounded up to 459.

### Sampling technique

Multi-stage sampling technique was employed. Selection of households was facilitated by obtaining a base map of Enugu State where all the slums were highlighted by the researcher and divided same, based on the local government areas in the metropolis which were clearly and serially identified. More so, the immunization records of the slums were also useful, especially in the use of their house numbering system. This involved serial stratification and selection.

In the first stage, there was the stratification of the Enugu Urban into 3 LGAs and the selection of one of them (Enugu North LGA). Stage 2 included stratification of the slums in the selected LGA and selection of two of them whereas in stage 3 selection of households was done using simple random sampling by balloting method, then selection of heads of households or the proxies.

### Study instruments

The study adopted the Housing and Health Questionnaire (HHQ) and an observation checklist. In the HHQ, housing conditions were measured using six broad variables: safety/security, indoor temperature controls/ventilation, hygiene/sanitation, building condition, environmental quality and crowding. The checklist was also adapted from HHQ. However, in the adapted version and the checklist, some of the variables were tailored to fit into our environment and the study aims. It was an interviewer-administered questionnaire with heads of household or a proxy respondent for the household. A pilot study was conducted in a non-selected slum to test the tool and validate it. The study stool was validated by the supervisor. The limitations of the tool were noted and the appropriate corrections were made and incorporated in the tool before the actual data collection.

### Data collection

Data was collected with the help of four (4) research assistants. The data collection lasted for 4 weeks. These research assistants were trained rigorously for 1 day on the research objectives and the study instruments. The session lasted for over four hours and they were made to recall and demonstrate the contents of the study tool until satisfaction was achieved.

### Data analysis

The data was entered and analysed using the IBM Statistical Package for Social Sciences (SPSS) version 23. A dichotomous indicator with zero (0) denoting the absence (no) of the element and one (1) indicating its presence (yes) was adopted for measurements.

The independent variables were socio-demographic variables, the housing conditions whereas the dependent variables were the reported health conditions and knowledge derived from the responses from the questionnaire. Categorical data were summarised with frequency and proportion whereas the continuous data were summarised using means and standard deviation. The total knowledge variables were rated and the proportion of those with good knowledge was noted. Good knowledge score was determined by having greater than 50% total score and above and poor level was < 50% score. The Chi-square test was used to assess association and the significance level was set *p* ≤ 0.05.

### Ethical consideration

The ethical clearance was obtained from the Health Research Ethics Committee (HREC) of the University of Nigeria Teaching Hospital Ituku-Ozalla, Enugu which is in the network of the National Health Research Ethics Committee (NHREC). Informed Voluntary Consent was also obtained from the household respondents after describing the aim of the study and anyone who wished to withdraw from the study was allowed to do so. Voluntary participation was ensured. Also, confidentiality was promised and maintained in the course of the study and even beyond.

### Limitations

The study was prone to recall bias since the respondents depended on their power of recall to attempt several variables. However, to circumvent this, each question was severally asked.

## Results

There was a response rate of 98.7% with 459 out of 465 questionnaires completed and returned.

The mean (SD) age of the respondents was 32.93(12.34) years and most of them (*N* = 180, 39.2%) were in the age range of 18–27 years. Two hundred and eighty-four (61.4%) of them were females whereas two hundred and ninety-seven (64.7%) were married. Most of them (*N* = 273, 59.5%) attended secondary school, however only a few (*N* = 9, 2.0%) did not access formal education. These respondents were predominantly Christians (*N* = 456, 99.3%), and Igbo (*N* = 453, 98.7%) with the most of them being self-employed (*N* = 327, 71.2%). Table 1.

**Table 1:** Socio-demographic characteristics of the residents of Enugu Urban slum in Enugu metropolis, South East, Nigeria

Variables	Frequency (*n* = 459)	Percentage (100)
**Age**		
18–27	180	39.2
28–37	153	33.3
≥ 38	126	27.5
***Mean(SD)***	***32.93(12.34)***	
**Gender**		
Male	177	38.6
Female	282	61.4
**Marital Status**		
Married	297	64.7
Single	162	35.5
**Educational Status**		
Informal	9	2.0
Primary	102	22.2
Secondary	273	59.5
Tertiary	75	16.3
**Religion**		
Christianity	456	99.3
Moslem	3	0.7
**Tribe**		
Igbo	453	98.7
Hausa	6	1.3
**Occupation**		
Unemployed	78	17.0
Self-employed/artisan	327	71.2
Employed by government	54	11.8
		,

Almost half of them (*N* = 231, 50.3%) had good knowledge of standard housing specifications whereas two hundred and ninety-seven (64.7%) of them rightly knew the effect of housing conditions on human health and source of knowledge was mostly via the television (*N* = 264, 57.5%) whereas the least source was from other sources like internet, etc. Most of these participants (*N* = 201, 43.8%) live in a room apartment but none lived in a bungalow or duplex. Table 2.

**Table 2:** Knowledge of unsafe housing conditions as contained in public health laws among the residents of Enugu Urban slum in Enugu metropolis, South East, Nigeria

Variables	Yes	No
	Freq(%)	Freq(%)
**Knowledge of standard housing specifications**	231(50.3)	228(49.7)
**Knowledge of the effects of housing conditions on human health**	297(64.7)	162(35.3)
**What are the sources of the knowledge?**		
Environmental health officer	180(39.2)	279(60.8)
Television	264(57.5)	108(23.5)
Radio	180(39.2)	279(60.8)
Public health lecture	138(30.1)	321(69.9)
Others	33(7.2)	426(92.8)
**The type of housing structure being occupied.**		
One-room apartment	201	43.8
Two-room apartment	81	17.6
Three-bedroom Flat	177	38.6
Bungalow	0	0.0
Duplex	0	0.0

On the availability of basic housing requirements/structural attributes of healthful housing among the respondents’ houses, the place of cooking as reported by most of them, (*N* = 150, 32.7%) was a separate kitchen and a few (*N* = 18, 3.9%) cooks were at open spaces. Heat supply for cooking as described by the majority was derived from gas cooker, (*N* = 249, 54.2%) and the least proportion was using the electric cooker (*N* = 27, 5.9%). Most of these slum dwellers use pour-flush toilets (*N* = 165, 35.9%) whereas sixty-three (13.7%) utilize other types of toilet facilities like open defecation. Most of these respondents used to stand and pour (*N* = 429, 93.5%) as their bathroom facility but three respondents each had a bathtub (0.7%) and no bathroom facility (0.7%) respectively. Considering the main source of water supply, most of them (*N* = 171, 37.3%) use pipe-borne water, whereas very few resort to using the services of water vendors, (*N* = 36, 7.8%) to source water. Four hundred and forty-seven (97.4%) use electricity as the source of light and three (0.7%) persons each use candles and kerosene lamps as the main source of light. Table 3.

**Table 3:** Availability of basic housing requirements/structural attributes of healthful housing among the residents of Enugu Urban slum in Enugu metropolis, South East, Nigeria

Variable	Frequency (*n* = 459)	Percentage (100)
**Place of cooking**		
Separate kitchen	150	32.7
Outside Kitchen	123	26.8
Bedroom	39	8.5
Corridor	129	28.1
Open space	18	3.9
**Sources of heat supply for cooking**		
Gas Cooker	249	54.2
Firewood	48	10.5
Electric Cooker	27	5.9
Kerosene Stove	33	7.2
Charcoal	102	22.2
**Toilet facility**		
Pour flush	165	35.9
Pit latrine	84	18.3
Water closet	147	32.0
Others specify	63	13.7
**Available Bathroom facility**		
Bathtub	3	0.7
Shower	24	5.1
Stand and pour	429	93.5
None	3	0.7
**The main source of water supply**		
Pipe borne water	171	37.3
Stream	54	11.8
Hand-dug well	84	18.3
Borehole water	45	9.8
Water vendors	36	7.8
Tanker water	69	15.0
**The main source of light**		
Kerosene lamp	6	1.4
Generator	3	0.7
Electricity	447	97.4
Candle	3	0.7

The availability of basic housing requirements and structural attributes of healthful housing among the respondents’ houses shows that the common sources of noise in the environment were traffic mainly, (*N* = 339, 73.9%); generators, (*N* = 150, 32.75%), church (*N* = 123, 26.8%). Most of the occupants were two persons per room, (*N* = 132, 28.3%), more than four per room (*N* = 99, 21.4%) but 15 of the slum dwellers live alone in a room. Three hundred and ninety (85.0%) of the respondents never renovated their apartments and more than four hundred and twenty (91.5%) had unkempt gutters in their surroundings. Table 4.

**Table 4:** Availability of basic housing requirements/structural attributes of healthful housing among the residents of Enugu Urban slum in Enugu metropolis, South East, Nigeria continues

Variable	Frequency (*n* = 459)	Percentage (100)
	Yes (%)	No (%)
**Common sources of noise in the environment**		
Generators	150(32.7)	309(67.3)
Traffic	339(73.9)	120(26.1)
Sound system/public address	126(27.5)	333(72.5)
Church	123(26.8)	336(73.2)
Others	9(2.0)	450(98.0)
**Occupants per room**		
One	45	9.8
Two	132	28.3
Three	96	20.9
Four	87	19.0
More than four	99	21.4
**Renovates apartment**		
Yes	69	15.0
No	390	85.0
**Availability of unkempt gutters**		
Yes	420	91.5
No	39	8.5

On the perceived possible effects of poor housing on the health of the respondents, two hundred and fifty-eight (56.2%) respondents had a history of sickness six months before the study and all two hundred and fifty-eight (100.0%) complained of malaria symptoms whereas ninety-six (37.2%) of them had typhoid fever within the same period and three (5.8%) had skin diseases by then. Most of them had malaria/fever, (*N* = 447, 97.4%) as the prevailing health condition. The common disease vectors found in their dwellings were mostly mosquitoes, (*N* = 438, 95.4%), rats (*N* = 387, 84.3%) and the least reported were fire ants (*N* = 129, 28.1%). The majority of the respondents use patent medicine dealers (chemists) as their source of primary care and a few (*N* = 18, 3.9%) use the PHC and thirty-six (7.8%) utilize the herbal medicine. Table 5.

**Table 5:** Perceived possible effects of poor housing on the health of the residents of Enugu Urban slum in Enugu metropolis, South East, Nigeria

Variables	Frequency (*n* = 459)	Percentage (100)
	Yes (%)	No (%)
**History of sickness in the previous 6 months**	**258(56.2)**	**201(43.8)**
**The type of ailment**		
Malaria	258(100.0)	0(0.0)
Respiratory symptoms like Cough	54(11.8)	208(79.1)
Hypertension	9(3.5)	249(96.5)
Typhoid fever	96(37.2)	162(62.8)
Skin conditions	3(5.8)	255(94.2)
Diarrhoea/watery stool	15(5.8)	243(94.2)
**Prevailing health conditions/challenges encountered**		
Malaria /fever	447(97.4)	12(2.6)
Skin conditions	66(13.7)	393(85.3)
Eye conditions	15 (3.3)	441(96.1)
Others	9(2.0)	450(98.0)
**Common disease vectors found in the dwellings**		
Cockroaches	318(69.3)	141 (30.7)
Rats	387(84.3)	72(15.7)
Mosquitoes	438(95.4)	21(4.6)
House flies	147(32.0)	312(68.0)
Fire ants	129(28.1)	330(71.9)
Termites	177(38.6)	282(61.4)
**Source of primary care**		
Chemist	375(81.7)	84(18.3)
Hospital	126(27.5)	333(72.5)
PHC	18(3.9)	441(96.1)
Trado/Herbal medicine	36(7.8)	423(92.2)

**Fig. 1:**
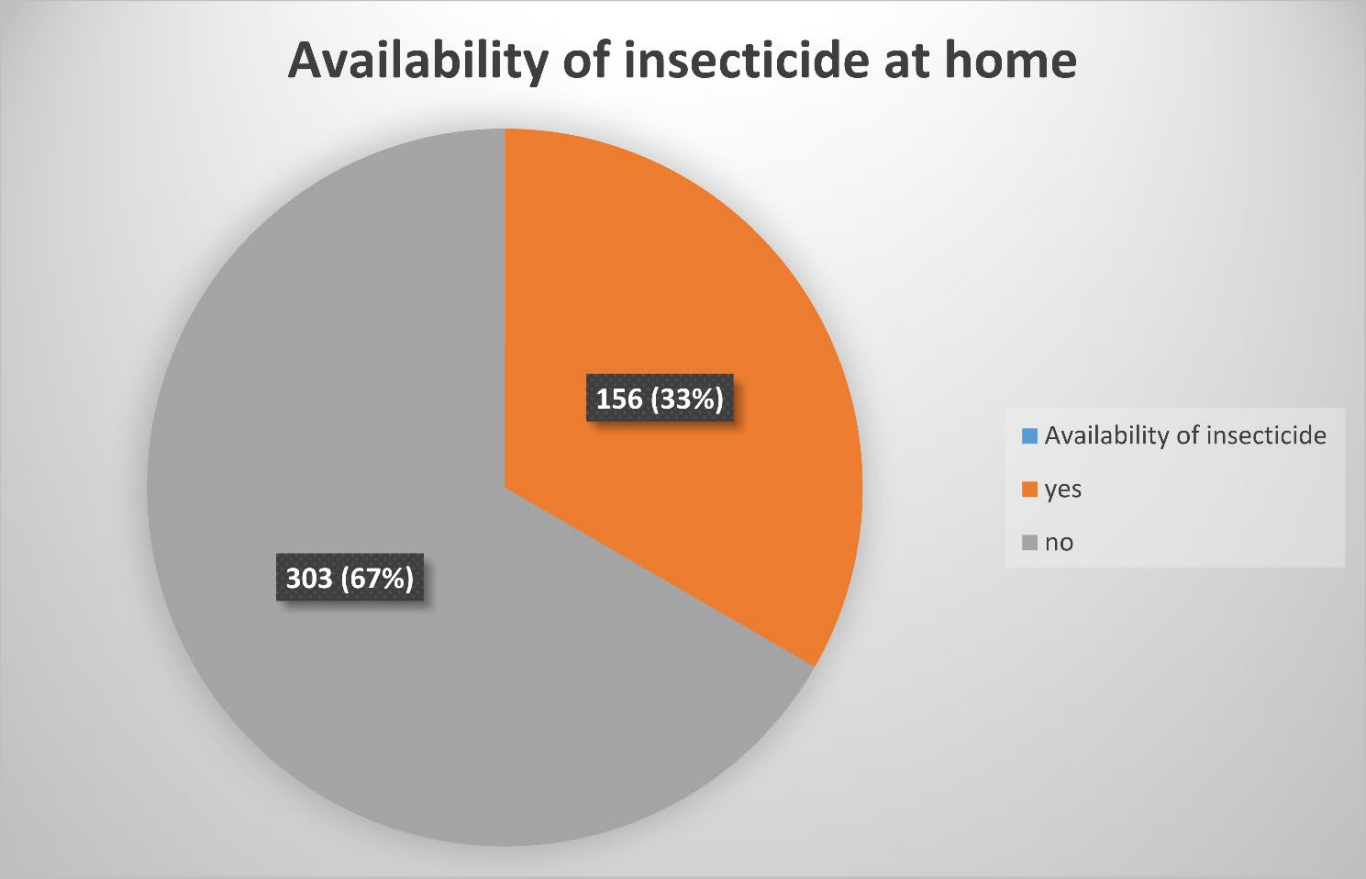
The availability of the insecticide treated nets among the residents of Enugu Urban slum in Enugu metropolis, South East, Nigeria

On the availability of insecticide at home as reported by the respondents, the majority of them did not have anyone (*N* = 303, 66.0%). Figure 1.

Generally, two hundred and fifty-five (55.6%) of their houses were adjudged to have adequate space and only a few (*N* = 51, 11.1%) of them had complete plumbing facilities which is defined as hot and cold piped water, a bathtub or shower and a flush toilet. Observing the dwellings, two hundred and forty (52.3%) of them were damp, two hundred and sixty-one (56.9%) of them were ill-ventilated, two hundred and seventy-six (60.2%) were littered with refuse, most, (*N* = 387, 84.4%) lacked important sanitary amenities and few of the dwellings, (*N* = 27, 5.9%) had facilities for prompt and sanitary solid waste disposal. Fire extinguishers were sighted in only twelve (2.6%) of the dwellings and eighteen (3.9%) had first aid boxes. Insecticide treated (mosquito) nets were observed in most of their houses (*N* = 294, 64.1%). Security dogs were also seen in thirty-two (20.9%) of the dwellings. Fans were also seen in four hundred and fourteen (90.2%) of the dwellings. The following poor building conditions were also noted: one hundred and thirty-two (28.8%) of the houses had broken or poorly done floors, and six (1.3%) had broken windows. Four hundred and twenty-three (92.2%) had toilet facilities and three hundred and twelve (68.0%) had evidence of mice/rat infestation. Table 6.

**Table 6:** Observation checklist assessment of healthful housing among the residents of Enugu Urban slum in Enugu metropolis, South East, Nigeria

Variable	Yes (%)	No (%)
Adequate Space	255(55.6)	204(44.4)
Complete plumbing facilities (defined as hot and cold piped water, a bathtub or shower, and a flush toilet.)	51(11.1)	408(88.9)
The dwellings for humans damp	240(52.3)	219(47.7)
Ill-ventilated	261(56.9)	198(43.1)
Dwellings littered with refuse	276(60.2)	183(39.8)
Dwellings lack important sanitary amenities	387(84.4)	72(15.6)
Houses have accessible roads	288(62.7)	171(37.3)
Drainage channels	237(51.6)	222(48.4)
Facilities for prompt and sanitary solid waste disposal	27(5.9)	432(94.1)
Regular and safe water supply	216(47.1)	243(52.9)
Dampness and mould growth	318(69.3)	141(30.7)
**Safety/security**		
Fire extinguisher	12(2.6)	447(97.4)
First-aid box	18(3.9)	441(96.1)
Mosquito net,	294(64.1)	165(35.9)
Fence wall	147(32.0)	312(68.0)
Security dogs	96(20.9)	363(79.1)
**Indoor temperature/ventilation**		
Fan,	414(90.2)	45(9.8)
Ceiling	216(47.1)	243(52.9)
Windows on two walls for cross-ventilation.	237(51.6)	222(48.4)
**Building condition**		
The condition of the roof: leaked	84(18.3)	375(81.7)
Wall: cracked	99(21.6)	360(78.4)
Windows (whether broken1	6(1.3)	453(98.7)
Ceilings: cracked	27(5.9)	432(94.1)
Floor (broken)	132(28.8)	327(71.2)
**Sanitation/hygiene**		
Toilet	423(92.2)	36(7.8)
Bathroom,	369(80.4)	90(19.6)
Potable water	21(4.6)	438(95.4)
Waste disposal facility	12(2.6)	447(97.4)
**Environmental Quality**		
Rainwater floods	240(52.3)	219(47.7)
Mice/rat infestation	312(68.0)	147(32.0)
Proximity of building to bush	186(40.5)	273(59.5)

A significant association between being sick, six months before the study and knowledge of the effects of housing conditions on health (Chi sq = 4.795; *p* = 0.029) exists but no significant association was noted with housing condition (Chi sq = 0.208; *p* = 0.648) or with knowledge of standard housing specifications (Chi sq = 0.644; *p* = 0.422) with sickness, 6 months before the study. Table 7.

**Table 7:** Association of housing conditions and knowledge with sickness in past 6 months among the residents of Enugu Urban slum in Enugu metropolis, South East, Nigeria

Variable	Sickness past 6 months		χ^2^ test	*p*-value
	Yes	No		
	*n*(%)	*n*(%)		
**Housing condition**				
Poor	180(55.0)	147(45.0)	0.208	0.648
Very poor	78(59.1)	54(40.9)		
**Knowledge of the effects of housing conditions on health**				
Yes	150(64.9)	81(35.1)	4.795	0.029
No	108(47.4)	120(52.6)		
**Knowledge of standard housing specification**				
Yes	174(58.6)	123(41.4)	0.644	0.422
No	84(51.9)	78(48.1)		

## Discussion

These slum dwellers exhibited good knowledge of housing and health however improving housing conditions in the homes is critically important because of its association with health. Sustainable Development Goals (SDG) 3 and 11 are directly related to health. While the latter focuses on sustainable cities and communities, the former emphasizes good health and well-being. Housing is not just a roof over one’s head but it is the conjunction of the dwelling, the home, the immediate environment and the community, in the contrary, housing is described as “inadequate” if it does not have basic facilities, infrastructure and services such as adequate space, ventilation, waste collection and disposal facility, sanitation, electricity, water supply and general environmental quality [10].

The majority of these respondents were within the age of 18 to 27 years with a mean of about 33 years. These findings were similar to that of the study done in Lagos State Nigeria [2, 24]. This shows that the slum inhabitants were mainly young people who were just starting independent living. This might also imply that these young people recently migrated from the village to the urban area or those that just finished serving their apprenticeship terms and were beginning to start independent living with little income, since rent is relatively cheaper in the slum. Most of them were married and this further strengthens the possibility of very young families. Most of these respondents were females. This was likely so because, at the time of the data collection, it was expected that the males would have gone to work to fend for the family.

Very few of them never had any education although, the majority were secondary school leavers. This level of literacy is quite commendable in a setting of this nature. A majority of them were self-employed and this was not unexpected since they are a relatively young population with secondary education.

About a half of the respondents exhibited good knowledge of standard housing specifications and the majority of them knew the effects of housing conditions on human health. This study is consistent with the findings in a similar study done in Lagos Southwest Nigeria where more than 75% of the study participants had good knowledge of the health effects of poor housing on humans [4]. Noteworthy was the slight difference in their proportions which could be attributed to the difference in the study area and study population, whereas this study was done in a slum where the majority of their highest educational attainment was noted to be secondary education and that of the Lagos study had tertiary education as their highest attainment. These findings further imply that these urban slum dwellers despite having good knowledge of the standard housing specifications and the impact of poor housing were constrained by their economic strength to seek a better alternative. Dimensions of health have gone beyond physical, mental, and psychosocial aspects including housing. Indoor air contamination, arising from cooking stoves fumes, mould and fungi growth, burning mosquito repellant, tobacco smoking and insecticide spray in the home showed significant effects on the respiratory symptoms (Cough, Wheezing, Pneumonia, Bronchitis and Asthma) among children [10].

Knowledge and evidence of the health risks in housing and the impact of interventions are vital to the development of policy and practice that tackles these risks. Lack of knowledge about the purpose and existence of health-promoting policies related to housing can be a barrier to policy implementation. The sources of their knowledge were mainly through, television, environmental health officer and radio. This implies that they commonly use the traditional news media where this information is usually shared.

The conditions in which people grow, live, work, and age contribute to their health and well-being. Housing inequalities have been established for ages and it has been shown that the differential place of living is closely linked to the socio-economic status of the dwellers [22]. While the housing policy empowers both individuals and organisations to own houses of their choice, it is pertinent to note that dwellers of both government-affordable houses and the slums can fall within the same socio-economic class [22]. This study also noted that none of the respondents lived in a bungalow or duplex but a majority lived in a room apartment where they share most facilities in common. Though, a substantial proportion reported living in a 3-bed room flat where some level of independence could be enjoyed. The places of cooking mainly were associated with in-door heating and air pollution leading to some respiratory infections.

Although, most of them cook with gas cookers, and use other methods like charcoal and kerosene stoves, firewood with very few using electric cook were also alternative sources. The implication of these methods remains worsening in-door air pollution which could cause and exacerbate respiratory problems and healthcare-related financial expenditure. Using a kerosene stove to cook in one room would amount to suffocation and devastating public health issues. This will affect the eyes, the nose and other vital organs and systems with a heavier impact among those having asthma and other chronic respiratory challenges.

The toilet facility as noted in this work was mainly a pour flush, water closet, and pit latrine. It will also be very noteworthy that some of them still adopt the unhygienic method to dispose of the toilet content. The public health implication of this can be very surprising as any event of disease outbreak in the slum, will spread like wide fire. This should be discouraged. Water sanitation and hygiene could not be professionally observed in this kind of setting.

The bathroom facility mostly was stand and pour type. Other modern bathroom facilities were missing. The study further observed that pipe-borne water was their major source of water supply. Some also use streams especially those living at the bank of the valley where seasonal water flows. These various water sources showed that water remains an essential facility. In terms of purity, wells and streams were most impure with contaminants. It was further noted in this study that they variously cook in separate kitchens, outside kitchens, corridors, bedrooms, and open spaces. Cooking in a separate kitchen may reduce indoor air contamination which will have less effect on the respiratory health of the inhabitants.

Many diseases have been linked to poor housing conditions. It has been shown that people with poor health and negative well-being were more likely to live in poor housing and conversely that improving housing conditions had also improved health and saved money [24]. The majority of these participants had reported being sick six months before this study and a majority of the complained ailment was malaria and typhoid fever as more prevailing health conditions. These findings were further supported by an Akure study on housing, where the predominant reported illness was malaria and typhoid fever [25]. Surprisingly, this development could not be linked with the place of dwelling since this Akure research was in an urban setting, hence these disease conditions were more environmentally-related and endemic in our Nigeria. A relatively clean environment could not salvage the urban city neither could clean water sources found in the urban could as well change this narrative. More so, people mix up, travel from one place to another even move with the vectors causing these health conditions. Very few of these dwellers reported skin infections as against the general background thinking that poor housing conditions would be associated with a high rate of skin infections [26], especially among children and the elderly [26]. Non-affirmation of this maxim in this research work, could be linked to a comparatively low sample size of these respondents. More so this study could not establish this kind of association or causality since it is not a case-control study or cohort study. Skin infection could be very irritating and worse still in an unhealthy environment. The low prevalence could as well be attributed to improved self-hygiene not minding the low practice of water, sanitation and hygiene. Several pathways have been demonstrated by some scholars on the link between housing and health and one of these pathways centres on the role of the neighbourhoods/environment (neighbourhood pathway) where whereas others focus on stability, safety and quality, and lastly the affordability pathways [27]. Common disease vectors as identified in this study were cockroaches, rats, flies, mosquitoes etc. These vectors were usually involved in varying disease causation pathways. The mosquitoes, malaria vector were ubiquitous in the study environment. Bites of others, like termites and, fire ants which were also reported can cause an irritable state.

Most of these dwellers sourced their healthcare services from patent medicine vendors (chemists) and the majority were noted as not using hospital facilities as part of their health-seeking behaviour. This is dissimilar to the findings done in Akure urban where the authors noted that the inhabitants seek healthcare mainly from the orthodox sources [25]. The difference in the findings most likely would be associated with the study area. The Akure study was carried out in the urban where the socio-economic status of the inhabitants was relatively higher. Buttressing the healthcare-seeking behaviour of the inhabitants of urban dwellings, a comparative study demonstrated that the use of outpatient primary care was significantly higher compared to the rural and suburban areas [27]. However, the implication of this approach to health care among the majority of the households remains in the reception of substandard healthcare services from the untrained healthcare personnel especially the PMV and other quacks. This will on an extended scale, impact the healthcare indices of the state and the nation at large. This will also on an individual basis affect their daily activities.

Overcrowding is defined using different measures that need to be culturally appropriate. Frequently used measures are, for example, the UN-Habitat definition of more than three people per habitable room and the Canadian and European definitions based theirs on single-person bedrooms (with exceptions for couples and young children/same-sex children) [11]. Given these context-dependent definitions, it is not surprising that space requirements and associated policy mechanisms to achieve housing and provide adequate living space also vary internationally. More than half of these inhabitants are already living in an overcrowded setting. Overcrowding is linked with an increased risk of epidemic-prone diseases like tuberculosis, respiratory tract infections, diarrhoeal diseases, cholera, and infectious hepatitis, mental health concerns, increased risk of accidents, and limited access to health care, social and economic challenges. They are the outcomes of poor sanitation and hygiene,

The study also revealed that most of the sampled households were inadequate and unhealthful. These findings were in tandem with the results of the study done in Akwa-Ibom, State, South-South Nigeria where the studied houses were characterised by poor housing facilities [10]. The impact of this finding on the household is that the majority of them live in an environment where the house poses a great challenge to their health since most of these shelters lack the facilities necessary to promote occupants’ health and wellbeing. These findings further highlighted the need for immediate action on housing intervention as they suggest that health among the slum dwellers could be strengthened with improved housing conditions since housing remains one of the social determinants of health [28].

Housing is not just a roof over one’s head but it is the conjunction of the dwelling, the home, the immediate environment and the community, in the contrary housing is described as “inadequate” if it does not have basic facilities, infrastructure and services such as adequate space, ventilation, waste collection and disposal facility, sanitation, electricity, water supply and general environment. The observation checklist showed that a substantial proportion of the houses had dampness and mould growth. This is unhealthy for man. Living in homes that were damp and mouldy increased the risk of experiencing health problems such as respiratory infections, allergic rhinitis and asthma and the presence of mould in buildings could pose severe health risks to babies, young children, elderly people, those with skin diseases and the people undergoing chemotherapy [25]. The most susceptible among these groups were children.

In another revelation, some multiple and complex relationships with poor housing have been established with HIV/AIDS as some authors have attempted to describe the link between poor housing conditions, HIV and AIDS as multiple and complex [25]. High densities, overcrowding and housing conditions increase the risk of opportunistic infections among these groups, and inadequate water and sanitation increase the probability of being infected and pose challenges to the provision of home-based care for these patients [25]. Another author established that overcrowding and some other aspects of a poor home environment contribute to mental stress and reduce people’s sense of general well-being [7]. It was further observed that most of the sampled household has good sanitation and hygiene facilities. Surprisingly, waste disposal facilities and potable water were grossly inadequate.

There is an association between high indoor temperatures and some adverse health effects [8]. In many settings, informal housing contributes to multiple health risks, including exposures to poor indoor air quality, excess indoor temperatures, lack of sanitation, inadequate access to improved water, unsafe structures, fire and electrocution risks, infestations and exposure to vector-borne diseases. Respiratory infections and diarrhoea diseases are two major killers that have been linked to inadequate home and neighbourhood environments [1, 6, 29], and these slum dwellers are at risk of all these conditions.

It is established that some health disorders such as typhoid and paratyphoid fever, diarrhoea, dysenteries, cholera, hookworm, ascariasis, viral hepatitis, guinea worm diseases, schistosomiasis, genito-urinary tract infections and many other intestinal and parasitic infections could be contacted through poor toilet facilities which may be a breeding ground for harmful bacteria, viruses and parasites [25]. The public health impact of these situations on the ground as reported could arise from any breach in the hygiene. A disease outbreak within this slum could be disastrous because of these poor and inadequate housing facilities coupled with inadequate health care delivery facilities. Such diseases could be cholera, food poisoning, and all the water-borne diseases.

This research work generally demonstrated that some of the observed buildings had substandard superstructures like leaking roofs, cracked walls and ceilings, and broken windows and floors. The state of the houses was not compatible with health and life. This magnitude of this status as captured in a systematic review on housing and health has shown that poor dwelling structures remain a threat to life [27]. This is further supported by thus proving that some environmental factors within homes are linked with poor health and in-home exposure to lead (from house paint flakes) irreversibly damages the brains and nervous systems of children [27]. Substandard housing conditions such as water leaks, poor ventilation, dirty carpets, and pest infestation have been associated with poor health outcomes including respiratory health challenges.

Good indoor temperature and ventilation are essential needs for healthful housing. A good proportion of these respondents had fans, ceilings and well-placed windows for good ventilation.

A large number of interventional studies have demonstrated the potential for improving health through improved housing quality and safety and studies where asthma triggers were removed had repeatedly demonstrated health improvements with good ventilation systems in the dwelling [4, 27]. This may be the explanation for the low prevalence of respiratory diseases among our respondents even though it can be contended that disease causation is known to be multifactorial [30]. However, this study did not deliberate on the day-light penetration ratio of these homes. The neighbourhood remains part of the housing because what affects the neighbourhoods can be a fatal threat to the survivorship of an individual in better housing but shares neighbourhoods with poor dwelling arena. The presence of floods, mice and rat infestation and proximity to the bush is partly red flags to some zoonotic outbreaks as well as outbreaks of water-related diseases, especially the diseases that can be worsened by flooding like food poisoning, diarrhoeal diseases (cholera, dysentery. etc.), skin diseases, and faecal-oral infections. This flooding may be related to poor drainage systems. Choked gutters and drains serve as breeding sites for mosquitoes with dengue fever and malaria transmission levels very high in slum settlements. Aedes mosquito, the vector for dengue fever, for instance, is adapted to slum conditions, in contrast to Anopheles mosquito, which thrives in high sunlight and plentiful vegetation.

Cholera is frequently reported in slums, especially during the rainy season and is compounded by the choked gutters and drains in these settlements. Typhoid transmission levels are very high in slums due to poor hygiene practices of food vendors.

The influence of physical surroundings had been noted as early as science is known as described by John Snow’s work on cholera and water pump handles [31]. Some studies have shown that being without a stable home is detrimental to one’s health [8, 10, 15, 32]. People who are chronically homeless face substantially higher morbidity in terms of both physical and mental health [27].

This study never assessed the affordability of urban housing, nor did it consider the willingness and desire to migrate out of the slum. Their quality of life as well as their fulfilment was not subjected to checks during this research and is open for further study.

## Conclusions

The evidence on the relationship between housing and health is complex but compelling. This study has shown that the household dwellers of the slum live in an environment short of the standards prescribed by WHO [11, 33–35]. With demonstrated good knowledge of standard housing specifications and good knowledge of the interrelationship between housing and health, they were relatively overcrowded and community noise of traffic origin was also identified as a major nuisance.

Continuous public health enlightenment among the slum dwellers especially on the health implications of their living conditions concerning their water, sanitation and hygiene is advocated. Further research should also be considered to assess the safety of indoor air quality levels among these slum residents and their association with cardiovascular and respiratory diseases including pneumonia, stroke and lung cancer.

## Electronic supplementary material

Below is the link to the electronic supplementary material.

Supplementary Material 1

## Data Availability

The data is available on request.
