# Supplementary material for: Urban slum housing quality, and its public health implications in Nigeria: a case of urban slum residents in Enugu metropolis, South East, Nigeria
Source: BMC Public Health. 2024 Nov 20;24:3231. doi: 10.1186/s12889-024-20764-7 (PMC11580511; doi:10.1186/s12889-024-20764-7)
Supplement: Supplementary file 1 — Supplementary Material 1 [file 12889_2024_20764_MOESM1_ESM.docx]

**Adapted Housing and Health Questionnaire**

**Socio-demographic variables**

1. Age as at last birth day [ ]
2. Sex: 1. Male [ ] 2. Female [ ]
3. Marital status: married [ ] single [ ] separated [ ] divorced [ ] widow/widowed [ ]
4. Educational level/status: primary [ ] secondary [ ] tertiary [ ]
5. Religion: Christianity Moslem African Trad religion others specify
6. Tribe: 1. Igbo [ ] 2. Hausa [ ] 3. Yoruba [ ] 4. Others (please specify)……………………….
7. Occupation: 1. Public servant [ ] 2. Self-employed [ ] 3. Artisan [ ] 4. Unemployed [ ]
8. Place of residence.( please specify)…………………..
9. Minimum daily water use……………….litres

Section A Safety/Security, indoor temperature controls/ventilation, hygiene/sanitation, building condition, environmental quality and crowding

**Section A: Knowledge of unsafe conditions of a house as contained in public health laws**

1. Are you aware of standard housing specifications ? 1. Yes [ ] 2. No [ ]
2. Are you Aware of the effects of housing conditions on health/ aware that conditions of housing affect the health of the residents.? a. yes [ ] b. no [ ]
3. What are your Sources of awareness? A. environmental health officer [ ] b. television [ ] c. radio [ ] d. public lecture [ ] others specify…….
4. How does housing affect your health?
5. What Type of housing structure do you live? A. one room apartment [ ] wo room [ ] flat [ ] others specify [……………….

**Section B: BASIC HOUSING REQUIREMENTS/Structural attributes of housing:**

1. Where do you cook food? A. separate kitchen [ ] b. kitchen outside the house [ ] c. bedroom [ ] d. corridor [ ] e. open space [ ]
2. What is your main Source of heat supply for cooking. A. kerosene stove b. gas cooker [ ] c. firewood [ ] d. charcoal [ ] e. electric cooker [ ] f. saw dust [ ]
3. What is your method of toilet facility? A. pour flush [ ] b. pit latrine [ ] c. water closet [ ] d. none [ ]
4. What type of bathroom facility do you have? A. bath tub [ ] b. shower [ ] c. stand and pour [ ] d. none [ ]
5. What is your main source of water supply? A. pipe borne water [ ] b. borehole water [ ] c. stream [ ] d. hand dug well [ ] e. water vendors [ ] f. tanker water [ ] g. sachet water [ ]
6. What is your main source of light? A. generator [ ] b. kerosene lamp [ ] c. electricity [ ] d. candle [ ] e. electricity and generator [ ] f. other sources: kindly specify……
7. Is there Availability of gutters? a. yes [ ] b. no [ ]
8. What are the common sources of noise? A . generators [ ] b. traffic [ ] c. sound system [ ] d. church/mosque [ ] e. others : kindly specify …………
9. How many of you stay in a room (number of people per room) ? a. one [ ] b. two [ ] c. three [ ] d. four [ ] e. others (specify) ……
10. Do you renovate your house

**SECTION C: Effects of housing on the health of residents**:

1. Have you been sick in the past six months? 1. Yes [ ] 2. No [ ]
2. Diseases reported in the past 6 months: Diseases mostly reported Malaria [ ] Cough [ ] Hypertension [ ] Typhoid fever [ ] skin conditions
3. **Prevailing health conditions/challenges:** fever/malaria [ ] skin conditions: skin rashes [ ]

Eczema [ ] Ring worm [ ] None [] eye conditions [ ]

1. **Common vector in dwellings** Cockroaches [ ] Rats [ ] Mosquitoes [ ] House flies [ ] fire ants [ ] termites [ ]
2. Source of primary care: chemist [ ] PHC [ ] hospital [ ]
3. Do you have insecticide treated mosquito net

**Appendix 2**

**Observation Check list:**

1. Adequate Space 1. Yes [ ] 2. No [ ]
2. Complete plumbing facilities (defined as hot and cold piped water, a bathtub or shower, and a flush toilet.) 1. Yes [ ] 2. No [ ]
3. Is the dwellings for humans damp,
4. Ill-ventilated 1. Yes [ ] 2. No [ ]
5. Dwellings littered with refuse. 1. Yes [ ] 2. No [ ]
6. Dwellings lack important sanitary amenities. 1. Yes [ ] 2. No [ ]
7. Houses should have accessible roads. 1. Yes [ ] 2. No [ ]
8. Drainage channels. 1. Yes [ ] 2. No [ ]
9. Facilities for prompt and sanitary solid waste disposal. 1. Yes [ ] 2. [ ]
10. Regular and safe water supply. 1. Yes [ ] 2. No [ ]
11. Dampness and mould growth

**Safety/security**: The presence or absence of

1. Fire extinguisher, 1. Yes [ ] 2. No [ ]
2. First-aid box 1. Yes [ ] 2. No [ ],
3. Mosquito net, 1. Yes [ ] 2. No
4. Fence wall 1. Yes [ ] 2. No
5. Security dogs 1. Yes [ ] 2. No

**Indoor temperature/ventilation,**

1. fan, 1. Yes [ ] 2. No
2. ceiling 1. Yes [ ] 2. No
3. windows on two walls for cross-ventilation. 1. Yes [ ] 2. No

**Building condition**,

1. The condition of the roof: leaked 1. Yes [ ] 2. No,
2. Wall: cracked 1. Yes [ ] 2. No [ ],
3. Windows (whether broken1. Yes [ ] 2. No [ ]
4. Ceilings: cracked 1. Yes [ ] 2. No
5. Floor (broken1. Yes [ ] 2. No [ ] .

**Sanitation/hygiene:**

1. Toilet 1. Yes [ ] 2. No [ ]
2. Bathroom, 1. Yes [ ] 2. No [ ]
3. Potable water 1. Yes [ ] 2. No [ ]
4. Waste disposal facility. 1. Yes [ ] 2. No [ ]

**Environmental quality:**

1. rainwater floods, 1. Yes [ ] 2. No
2. mice/rat infestation 1. Yes [ ] 2. No
3. proximity of building to bush. 1. Yes [ ] 2. No
